# Electrostatically Induced Intercalation of Layered Double Hydroxide in Graphene Oxide for Enhanced Electrochemical Energy Storage

**DOI:** 10.1002/advs.202515923

**Published:** 2025-10-05

**Authors:** Xiaojun Ren, Tongxi Lin, Bing Sun, Zeno Rizqi Ramadhan, Hang Yin, Furqan Hussain, Quanbin Dai, Richard Tilley, Liming Dai, Zi Gu, Rakesh Joshi

**Affiliations:** ^1^ School of Materials Science and Engineering University of New South Wales Sydney NSW 2052 Australia; ^2^ Australian Carbon Materials Centre (A‐CMC) School of Chemical Engineering University of New South Wales Sydney NSW 2052 Australia; ^3^ Mark Wainwright Analytical Centre University of New South Wales Kensington NSW 2052 Australia; ^4^ School of Minerals and Energy Resources Engineering University of New South Wales Sydney NSW 2052 Australia

**Keywords:** layered double hydroxide, reduced graphene oxide, supercapacitor

## Abstract

Graphene‐based materials have great potential for electrochemical energy storage applications, but their performance is often limited by the restacking of nanosheets, which restricts ion accessibility. In this study, a straightforward method to fabricate reduced graphene oxide (rGO) laminates intercalated with magnesium–aluminium layered double hydroxide (MgAl‐LDH) nanosheets is presented. Due to electrostatic interactions, the positively charged LDH nanosheets strongly bind to the negatively charged rGO layers, forming a stable, alternating laminar structure with well‐defined nano‐capillaries. Detailed characterization confirms the intended architecture of the rGO‐LDH hybrid. Electrochemical analysis shows nearly ideal electric double‐layer capacitor (EDLC) behavior, with the rGO‐LDH reaching a specific capacitance of up to 410 F g^−1^ at 1 A g^−1^. This work highlights the vital role of LDH nanosheets as interlayer spacers that effectively prevent restacking, providing new insights into designing 2D materials for high‐performance supercapacitors and energy storage systems.

## Graphical abstract

Electrostatically intercalated MgAl‐layered double hydroxide (LDH) nanosheets act as non‐redox spacers to prevent rGO restacking via a self‐assembly process. Such layer‐by‐layer structure provides accessible nano‐channels for rapid ion transport, exhibiting high capacitance (410 F g^−1^) with robust retention and cycling stability. This strategy provides a scalable method of designing the layered 2D materials for next‐generation supercapacitors.

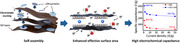

## 1 Introduction

Emerging 2D graphene‐based materials yield a variety of promising candidates for the next generation of energy storage systems,^[^ ^1^, ^2^, ^3^, ^4^, ^5^, ^6^, ^7^, ^8^, ^9^ ^]^ due to their high electrical conductivity, mechanical strength, and large specific surface areas.^[^ ^10^, ^11^ ^]^ Among all, graphene oxide (GO) has attracted widespread interest. The abundant oxygen functionalities on its surface enable further chemical modifications for various applications.^[^ ^12^, ^13^, ^14^, ^15^, ^16^, ^17^ ^]^ However, its low electrical conductivity limits its direct use for energy storage. Reduced graphene oxide (rGO) obtained through thermal or chemical reduction of GO can provide significantly improved conductivity on the surface by restoring an extended conjugated carbon network for electron transport after partially removing oxygen functionalities.^[^ ^12^, ^18^ ^]^ Yet, such a process may cause rGO to suffer from non‐reversible stacking of adjacent layers, result in the dramatic reduction of accessible surface of the material, further limiting its potential as electrodes for energy storage and catalysts.^[^ ^6^ ^]^

Numerous strategies have been developed to address such challenges. For example, molecules and nanoparticles can be inserted between rGO layers as spacers to prevent restacking and preserve a highly accessible surface.^[^ ^1^, ^19^, ^20^, ^21^ ^]^ The hybrid of rGO with other types of 2D materials in a layer‐by‐layer structure cannot only stop the restacking of rGO sheets but also create tunable 2D channels for rapid ion transport and conductivity. Layered double hydroxide (LDH), which consists of positively charged hybrid metal hydroxide layers separated by charge‐balancing anions, is a promising candidate for this purpose,^[^ ^1^, ^21^, ^22^, ^23^, ^24^, ^25^ ^]^ especially nanoparticles with atomic thin layers.^[^ ^26^, ^27^ ^]^ Since many reported studies of rGO/LDH hybrids rely on transition‐metal‐based LDHs (e.g., Ni, Co, Mn).^[^ ^28^, ^29^, ^30^ ^]^ These components mostly introduce faradaic redox reactions and thus battery‐like or pseudo‐capacitive behaviors with compromised power density and cycling stability, which are unfavorable for capacitor devices. However, LDH nano‐spacers with non‐transition‐metal composition may avoid such redox contributions, ensuring purely capacitive behaviors. Moreover, the positively charged surface of LDH can electrostatically interact with the negatively charged rGO surface, resulting in a continuous and spontaneous alternation between rGO and LDH layers.^[^ ^31^, ^32^, ^33^ ^]^

In this work, we proposed an electrostatically induced self‐assembly strategy to intercalate LDH nanosheets into rGO laminates, thereby achieving high‐performance electrochemical energy storage. Herein, we specifically synthesised LDH nanosheets based on non‐transition metals (magnesium and aluminium). This allows us to understand the enhancement of material electrochemical capacitive behaviours from the material structure point of view.^[^ ^34^, ^35^, ^36^ ^]^ We, through in‐depth chemical and physical characterisations and electrochemical measurements, found that the LDH nanosheets play a vital role as spacers inside rGO laminates to produce rGO‐LDH hybrids as a promising material for electrochemical‐based energy storage.

## 2 Results and Discussion

### 2.1 Electrostatically Induced Synthesis of rGO‐LDH

**Figure 1:**
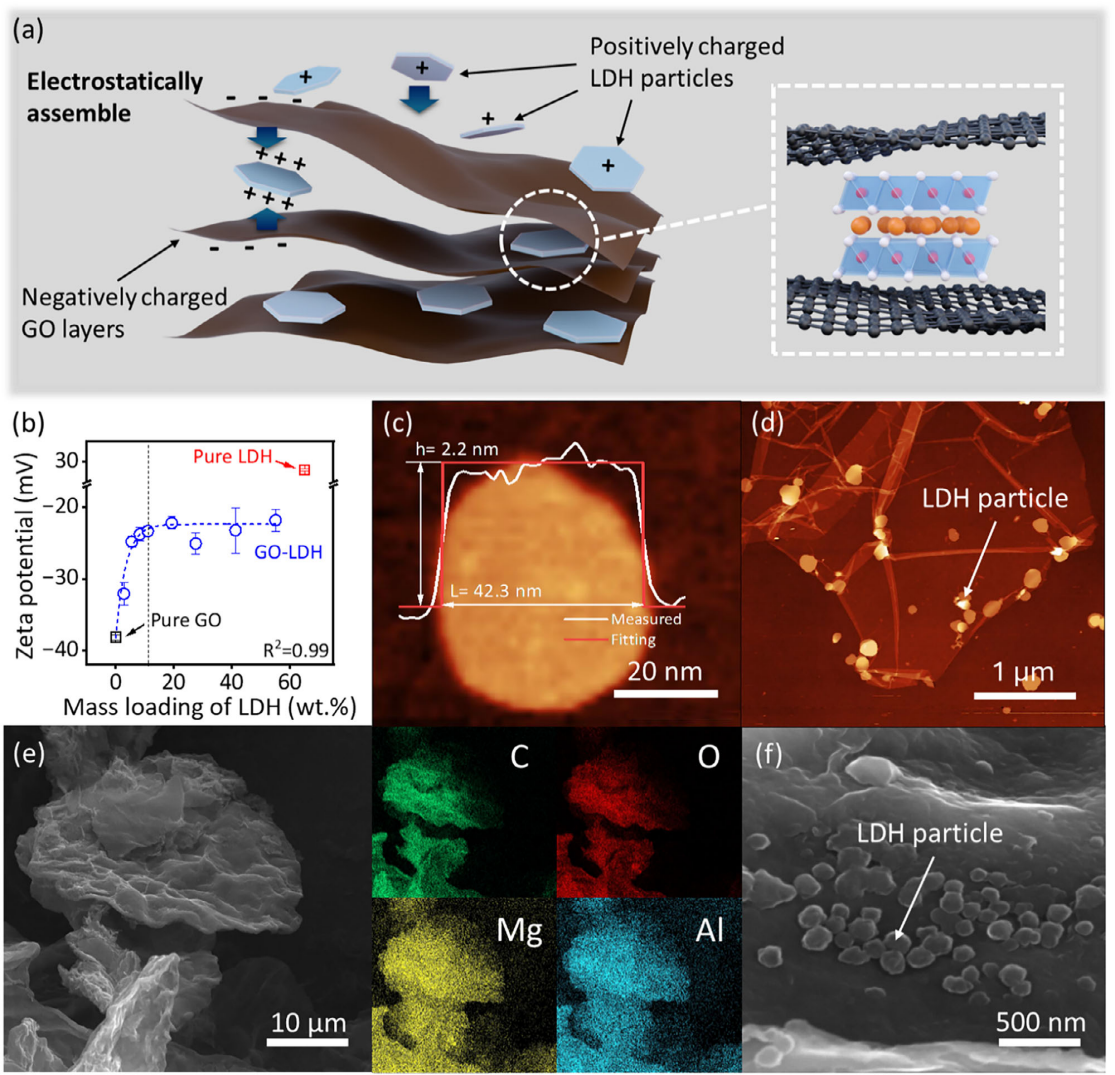
Electrostatic force‐induced intercalation of LDH nanoparticles. a) Schematic illustration of electrostatically induced intercalation of LDH nanoparticles on GO nanosheets. b) Zeta potential measurements of GO suspension mixing with various mass loadings of LDH nanoparticles. The vertical dashed line guides the eyes to the saturation point. The red square indicates the zeta potential of pure LDH water suspension. c,d) Atomic force microscopy (AFM) images of (c) a single LDH nanoparticle and (d) LDH particles intercalated with GO nanosheet. The inset diagram in (c) presents the height profile of the LDH nanoparticle. e,f) Scanning electron microscopy (SEM) images of rGO‐LDH with energy dispersive spectroscopy (EDS) showing elemental images of carbon, oxygen, magnesium, and aluminium.

We synthesized the layered double hydroxide (LDH)‐rGO hybrid laminates via electrostatically induced intercalation (**Figure** **1**a).^[^ ^37^ ^]^ To examine the electrostatic interaction between LDH and GO, we first confirmed the presence of surface charge on LDH particles and GO laminates in aqueous dispersion using zeta potential measurements. Our results show that the zeta potential of pure GO laminates is ≈−38 mV, indicating a negative charge, consistent with previous studies.^[^ ^33^, ^38^, ^39^, ^40^ ^]^ In contrast, LDH nanoparticles are positively charged with a zeta potential of +29 mV. The oppositely charged surfaces of LDH and GO sheets enable them to interact in water solution, leading to self‐assembly into LDH‐intercalated GO laminates (GO‐LDH). We measured the zeta potential of GO‐LDH with different mass ratios of GO to LDH. Notably, the zeta potential of GO‐LDH with ≈15 wt.% of LDH particles intercalated into GO laminates saturated at ≈−23 mV, as shown in Figure 1b. Since no further change in zeta potential was observed with additional LDH loading, we consider ≈15 wt.% of LDH to be the saturation point for intercalation. LDH particles, with an average length of ≈38 nm in diameter and a thickness of ≈3.3 nm (equivalent to 2–3 atomic layers), were confirmed via atomic force microscopy (AFM). Statistical data of LDH particles are provided in Note S1 (Supporting Information), and the AFM image of a single LDH particle is shown in Figure 1c as an example. Figure 1d displays the AFM image illustrating the morphology of the synthesized GO‐LDH, with LDH particles distributed on the surface of a monolayer GO nanosheet.

After intercalation, we freeze‐cast the GO‐LDH dispersion with a LDH mass loading of 15 wt.% to form the GO‐LDH hybrids, then thermally reduce them to rGO‐LDH. Scanning electron microscopy (SEM) images (Figure 1e,f) show the uniform intercalation of LDH particles within the rGO sheets. The rGO‐LDH samples display a highly wrinkled surface, similar to that of the GO‐LDH samples; however, they are notably different from the pure rGO samples. Additional SEM images are provided in Note S2 (Supporting Information). Energy–dispersive X‐ray spectroscopy (EDS) analysis confirms the presence of carbon, oxygen, magnesium, and aluminum as the main elemental composition of rGO‐LDH. This indicates a uniform distribution of LDH particles intercalated within the rGO nanosheets.

### 2.2 Composition and Structure of rGO‐LDH

**Figure 2:**
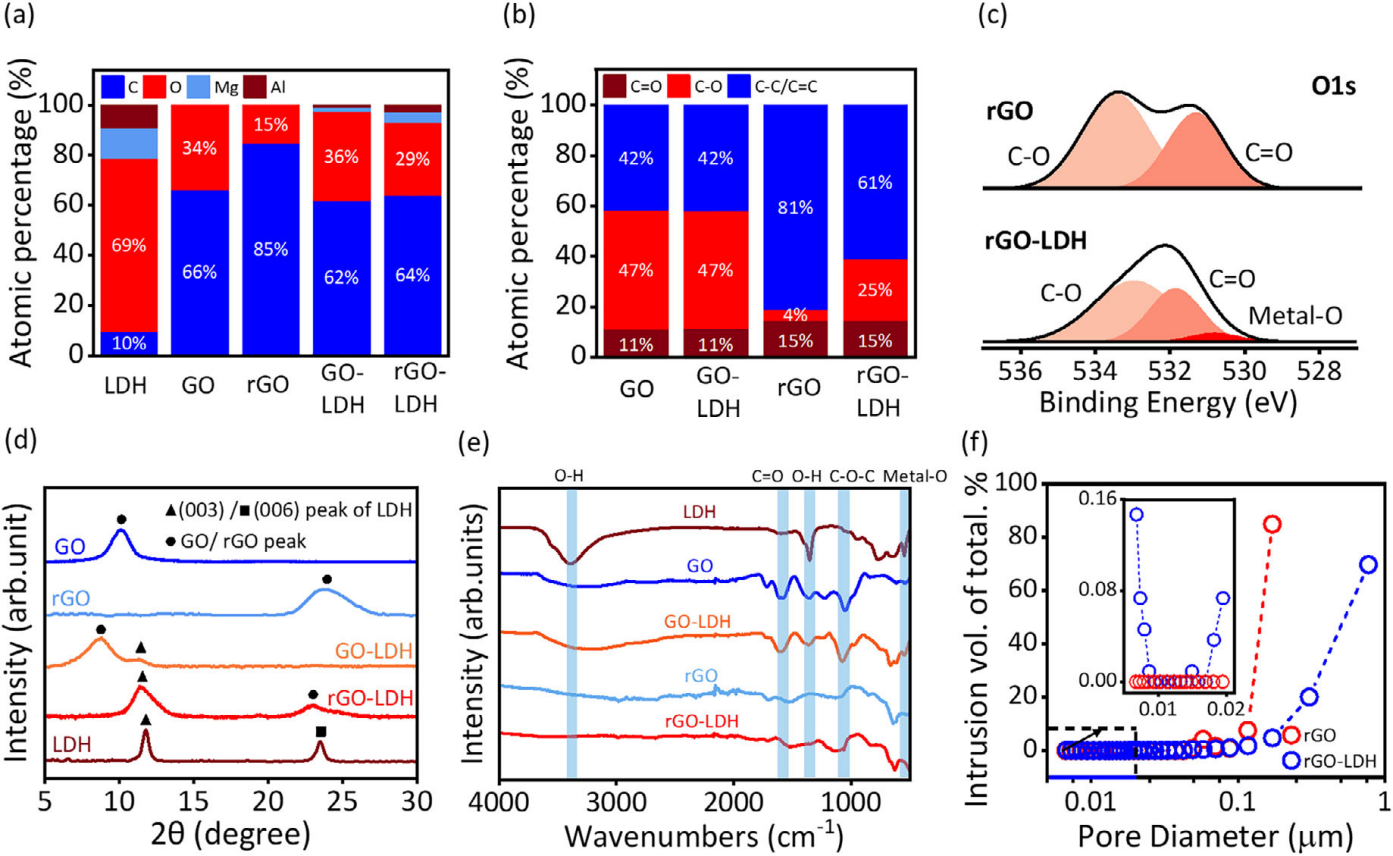
Characterisation of LDH nanosheets intercalated rGO. a,b) The atomic elemental a) and carbon bonds b) composition of LDH, GO, rGO, GO‐LDH, and rGO‐LDH, summarised from X‐ray photoelectron spectrometry (XPS) survey as detailed illustrated in Note S3. c) O1s XPS spectra of rGO and rGO‐LDH, with peak fitting of organic C═O bond (light orange) at ≈531.8 eV, organic C─O bond (light red) at ≈533 eV, and metal‐O bond (red) at ≈530.7 eV. d,e) X‐ray diffraction (XRD) patterns d) and Fourier‐Transform Infrared spectroscopy (FT‐IR) spectra e) of LDH, GO, rGO, GO‐LDH, and rGO‐LDH samples. Peaks in FT‐IR spectra at wavelength of ≈3420/1370, 1725, 1220, and 510 cm^−1^ (light blue area) represent the O─H, C═O, C─O─C, and Metal‐O stretching, respectively. f) Mercury injection capillary pressure (MICP) of rGO and rGO‐LDH samples, showing pore diameter from the range of 1 µm–3 nm. The blue arrow indicates the magnified image within the range of pore diameter from 3 to 25 nm.

X‐ray photoelectron spectroscopy (XPS) reveals changes in atomic composition during synthesis. The XPS survey scans of elemental composition are summarized in **Figure** **2**a, with detailed data provided in Note S3 (Supporting Information). The presence of Mg and Al, along with the increase of oxygen atomic percentage in GO‐LDH and rGO‐LDH, confirms the intercalation of LDH into GO nanosheets. The carbon detected in LDH might be due to the presence of CO_3_^2−^ anions in LDH interlayers.^[^ ^32^ ^]^ The XPS C1s peaks of GO and GO‐LDH show no significant difference, as shown in Figure 2b. This suggests that the intercalation between LDH particles and GO sheets is primarily electrostatic, rather than formed by new chemical bonds on the carbon atoms. Furthermore, Metal‐O bonds from LDH are detectable within GO‐LDH and rGO‐LDH samples in the XPS O1S spectra (Figure 2c). The rise in carbon percentage from GO to rGO and from GO‐LDH to rGO‐LDH confirms the reduction process post‐thermal treatment (Figure 2a). Analyzing the C1s spectrum, thermal reduction of GO‐LDH to rGO‐LDH leads to a significant decrease in C═O and C─O bonds (Figure 2b). Additionally, the obvious decrease of C─O bonds indicates that thermal treatment mainly removes hydroxyl groups from the GO basal plane rather than carboxyl groups at the edges.^[^ ^12^, ^15^, ^41^, ^42^ ^]^

X‐ray diffraction (XRD) analysis was conducted to examine the material structure (Figure 2d). The XRD pattern of GO displays a peak at 10.2° (*d* = 0.88 nm), representing the interlayer spacing between adjacent GO sheets. After intercalation, for GO‐LDH, this peak shifted left to 8.6 ° (*d* = 1.02 nm), with a new peak appearing at 11.3 ° (*d* = 0.78 nm), which is a typical (003) peak of the LDH nanoparticles. This shows that after intercalation, the average d‐spacing between GO sheets expanded to ≈1.1 nm with the intercalated LDH. After thermal reduction, rGO shows a peak at 23.8 ° (*d* = 0.38 nm), consistent with reported studies.^[^ ^41^, ^43^, ^44^, ^45^ ^,]^ Notably, rGO‐LDH displays both the (003) and the rGO peaks, indicating that the LDH particles not only stay within the rGO interlayer spacing but also retain their original crystal structure. Furthermore, pure LDH nanoparticles exhibit the same XRD pattern before and after thermal reduction, suggesting that the thermal treatment at the tested temperature may not cause phase changes in LDH (Note S4, Supporting Information). These findings provide direct evidence of the laminated structure of rGO‐LDH hybrids after intercalation and thermal reduction. The slight shift and separation of the rGO peak may be due to the strong electrostatic force between rGO and LDH nanoparticles. We also investigated the functional groups of our materials using the Fourier‐Transform Infrared spectroscopy (FTIR). As shown in the spectrum (Figure 2e), the broad peak at ≈3420 and the peak at ≈1370 cm^−1^ correspond to the O─H stretching of water.^[^ ^46^ ^]^ The distinct peak at ≈540 cm^−1^ represents the Metal‐O stretching.^[^ ^47^ ^]^ After intercalation, the intensity of both O─H and Metal‐O stretching is significantly enhanced in GO‐LDH, confirming the successful incorporation of LDH nanoparticles within the GO frameworks. Apart from these changes, the stretching vibration of other functional groups remains mostly the same, indicating that the GO‐LDH intercalation is dominated by electrostatic attraction rather than covalent bonding. After thermal reduction, rGO exhibits a remarkable decrease of O─H and C─O─C stretching bands, consistent with the removal of oxygen functionalities during the reduction process. Importantly, rGO‐LDH retains both Metal‐O and a weak O─H stretching signal, indicating the stability of the LDH inside rGO flakes. These results strongly support the electrostatic interaction between LDH and GO/rGO and also suggest the structural stability of rGO‐LDH hybrid after thermal treatment, which aligns with the XRD results discussed previously.

The porous structures of rGO and rGO‐LDH were investigated using mercury injection capillary pressure (MICP) analysis (Figure 2f). From a macrostructure perspective, the sub‐micrometre pores of rGO‐LDH hybrids are more accessible to mercury atoms than those of pure rGO sheets. The porosity analysis indicates that strong van der Waals forces between rGO laminates hinder the opening of interlayers, preventing mercury molecules from entering the interior under high intrusion pressures (up to 30 000 psia). However, in rGO‐LDH hybrids, the intercalation of LDH particles prevents the self‐stacking of rGO layers, enabling mercury atoms to enter these nanometer‐scale capillaries.

**Figure 3:**
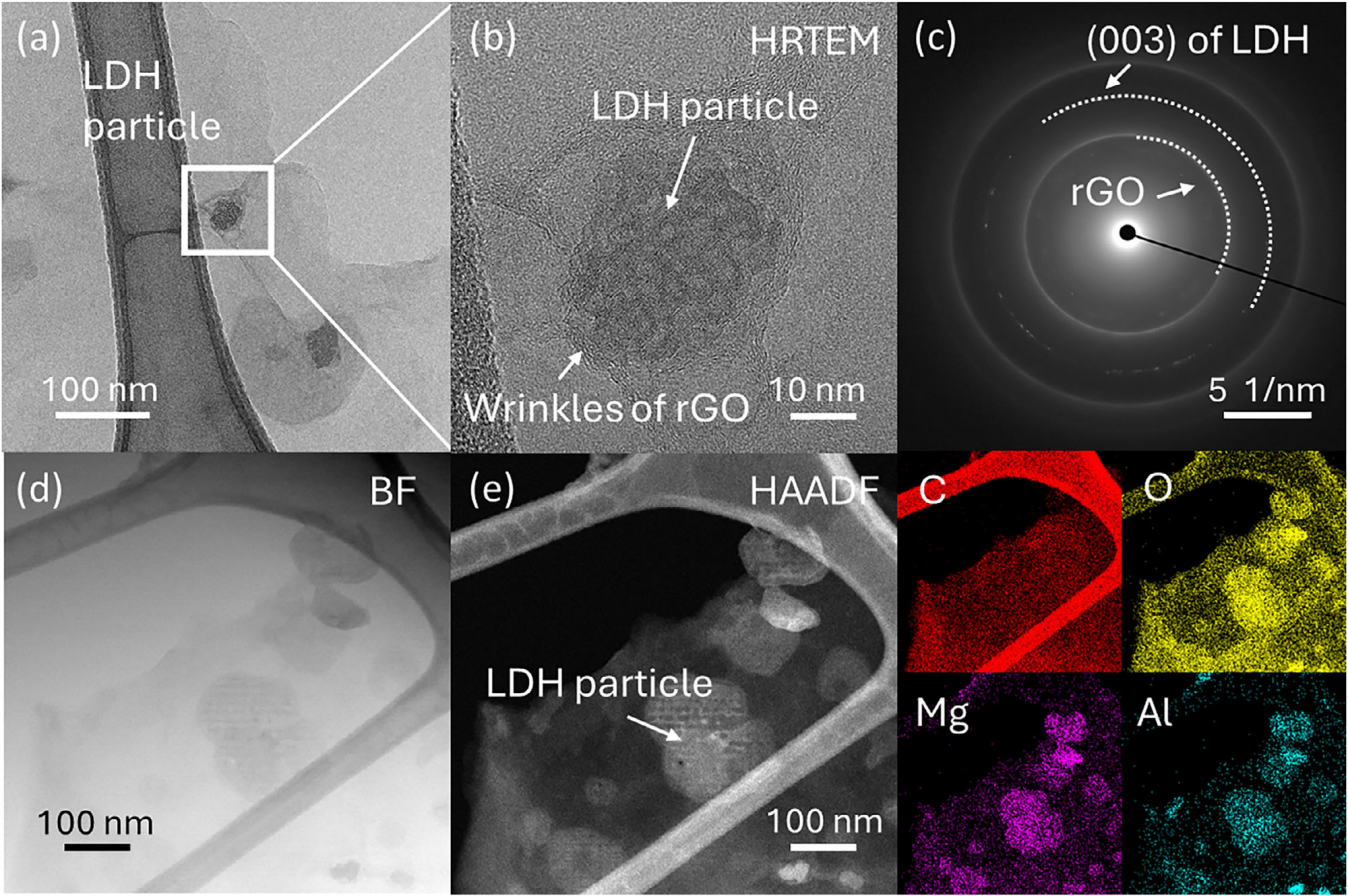
Transmission electron microscopy (TEM) images of rGO‐LDH. a,b) High‐resolution TEM (HR‐TEM) images of LDH particles on rGO nanosheets. c) Selected‐area electron diffraction (SAED) pattern of the highlighted area in (a). The semi‐circular dashed curve indicates the diffraction ring of rGO nanosheets (inner) and LDH particles (outer) at the (003) plane. d,e) Bright‐field (d) and high‐angle annular dark‐field (HAADF) (e) TEM image of rGO‐LDH hybrid nanosheets with energy–dispersive X‐ray spectroscopy (EDS) imaging of carbon, oxygen, magnesium, and aluminium.

The TEM image shown in **Figure** **3**a confirms the presence of LDH particles on the rGO nanosheets. Using high‐resolution (HR) TEM (Figure 3b), we observed the highly crystalline structure of the LDH particles, located near the wrinkles of the rGO sheets. We suggest that the formation of these wrinkles might also result from the strong electrostatic forces mentioned previously. The selected area electron diffraction (SAED) pattern, indicated as a white box in Figure 3a, displays a clear diffraction ring (Figure 3c) corresponding to the (003) plane of the LDH particles, along with a weak diffraction ring of rGO. This aligns with the XRD patterns presented earlier. To better understand the structure, we also examined the rGO nanosheets with LDH particles using high‐angle annular dark‐field scanning transmission electron microscopy (HAADF‐STEM), due to the different Z‐contrast from carbon and the magnesium and aluminium components of LDH. In the same area, the LDH particles are visible with high contrast in both bright‐field (Figure 3d) and dark‐field (Figure 3e) images. The energy dispersive X‐ray spectroscopy (EDS) mapping results confirm that the high contrast areas contain four elements: carbon, oxygen, magnesium, and aluminium. These findings confirm that LDH particles are closely aligned with the basal plane of the rGO sheets.

### 2.3 Electrochemical Profiles of rGO‐LDH

**Figure 4:**
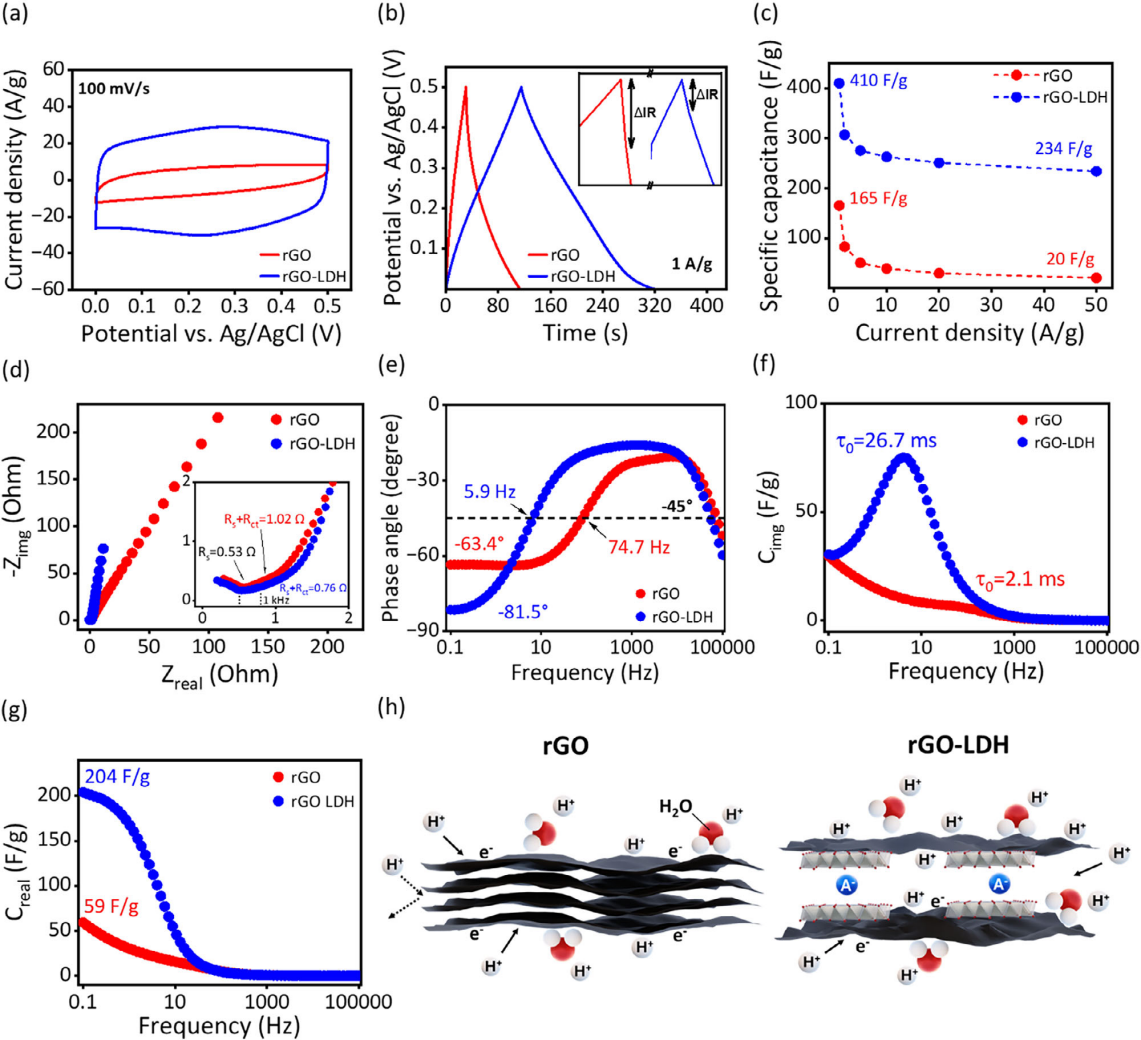
Electrochemical properties of rGO and rGO‐LDH in three electrode system. a) Cyclic voltammetry (CV) measurements within the range of potential from 0 to 0.5 V at the scan rate of 100 mV s^−1^. b) Galvanostatic charge–discharge (GCD) measurements at the current density of 1 A g^−1^. The inset diagram illustrates the magnified GCD curves showing the IR drops (ΔIR). c) Comparison of the specific capacitances under different current densities. d–g) Electrochemical impedance spectroscopy (EIS) measurements including Nyquist plots (d) and Bode plots (e–g). The inset diagram in d) illustrates the magnified image of Z_real_ from 0 to 2 Ω. h) Schematic illustration of ion transport mechanism via rGO and rGO‐LDH nanosheets.

To explore the potential applications of our materials synthesized through this intercalation method, we carried out comprehensive electrochemical measurements to study the electrochemical profiles of rGO‐LDH and compared them with samples made of pure rGO synthesized under the same thermal treatment conditions. The main results are shown in **Figure** **4**, with additional findings presented in Note S5 (Supporting Information). We compared the CV curves of rGO and rGO‐LDH measured at 100 mv s^−1^, revealing significant differences. In Figure 4a, the CV curve of rGO‐ LDH displays an almost ideal rectangular shape, along with a pair of broad and symmetric redox peaks ≈0. 25 V (vs Ag/AgCl electrode). This voltammogram indicates a combination of electrical double‐layer capacitance (EDLC) and pseudo‐capacitance behaviors.^[^ ^7^, ^36^ ^]^ It is important to highlight that the MgAl‐ LDH particles used in this work are free from transition metals, preventing electrochemical redox reactions. As mentioned, previous studies have shown that bulk LDHs containing transition metals typically display battery‐like redox behaviors. In contrast, MgAl‐LDH (non‐transition metal‐based) particles may exhibit more extrinsic pseudo‐capacitance due to large surface area, shortened ion diffusion paths, and fast surface redox for electrolyte ions.^[^ ^48^, ^49^, ^50^ ^]^ Its intrinsic pseudo‐capacitance is limited due to the lack of redox reactions, which is confirmed by the CV curve of bulk MgAl‐LDH (Note S5, Supporting Information). This is also consistent with our hypothesis that LDH particles are primarily acting as interlayer spacers within rGO sheets. Therefore, the pseudo‐capacitance observed may mainly arise from Faradic reactions linked to the remaining oxygen functionalities on the rGO basal planes.^[^ ^51^, ^52^ ^]^ Compared to rGO‐ LDH, the CV curves of rGO samples show a slightly distorted rectangular shape with much lower current density at the same potential, also accompanied by a pair of broad, symmetric redox peaks. The distorted rectangular shape (Figure 4a) suggests slower proton transport on the rGO surface, reflecting a higher equivalent series resistance (ESR) of the electrode.^[^ ^36^ ^]^ Simultaneously, the lower current density indicates a significantly smaller effective surface area for surface ion adsorption on rGO electrodes. This observation closely aligns with the porous structure seen via MICP as previously described. To better understand the diffusion and capacitive processes during the measurements, we also examined the capacitance contributions of rGO‐LDH and rGO electrodes using CV curves based on Dunn's method.^[^ ^53^ ^]^ The capacitance contribution of rGO‐ LDH electrodes exceeds 90% at a low scan rate of 10 mv s^−1^, whereas for rGO, it is only 39% under the same conditions. These findings are detailed in Note S6 (Supporting Information).

We investigated the charge‐discharge process of both electrodes at a low current density of 1 A g^−1^ using the galvanostatic charge‐discharge (GCD) method (Figure 4b). Both electrodes exhibit nearly ideal triangular charge‐discharge curves, indicating EDLC is the main mechanism of charge storage.^[^ ^36^ ^]^ The symmetry of the charge and discharge profiles of the rGO‐LDH electrode suggests good reversibility and stable capacitor performance. The specific capacitance of the rGO‐LDH electrode was estimated to be ≈410 F g^−1^, surpassing that of the pure rGO electrode (165 F g^−1^). The internal resistance of the electrodes is shown by the voltage drop at the start of discharging, known as the IR drop. The rGO electrode exhibits a more pronounced IR drop compared to the rGO‐LDH electrode, as shown in the inset of Figure 4b, indicating higher internal resistance. This agrees with the higher ESR observed in the CV curves of the rGO electrode. Conversely, the much lower IR drop for the rGO‐LDH electrode reveals its lower internal resistance. This may be due to the higher hydrophilicity of LDH particles on the rGO basal plane, which enhances interaction with the electrolyte and improves ion transport. The rate capability of the electrodes is shown in Figure 4c. The rGO‐LDH electrode achieves the highest specific capacitance at 1 A g^−1^ and retains 56.8% of its capacity when the current density increases to 50 A g^−1^. In comparison, the rGO electrode shows a much lower retention of 12.6% at 50 A g^−1^.

Electrochemical impedance spectroscopy (EIS) offers deeper insights into ion transport behaviors via electrodes. The Nyquist plots are shown in Figure 4d. The rGO‐LDH electrode exhibits a more vertical curve in the low‐frequency region, indicating closer‐to‐ideal capacitive behavior.^[^ ^36^ ^]^ In the high‐frequency region, the rGO‐LDH electrode displays a smaller interfacial resistance of ≈0.76 Ω compared to the rGO electrode, which measures 1.02 Ω. This aligns with our IR drop observation from the GCD measurement, pointing to faster charge transfer and improved interfacial ion transport at the rGO‐LDH surface. The Bode phase angle plot in Figure 4e further supports these findings. At 0.1 Hz, the phase angle of rGO‐LDH is significantly more negative (−81.5 °) compared to rGO (−69.4°). Additionally, rGO‐LDH electrodes show a higher relaxation time constant (τ_0_) of 26.7 ms vs 2.1 ms for rGO, as shown in Figure 4f. This may be due to the different porous structure of the two materials. In stacked rGO, the accessible surface area is mainly limited to external surfaces, restricting ion‐transport to short diffusion pathways, which only requires very limited time. However, in the rGO‐LDH hybrid, the intercalation of LDH nanosheets provides significantly more interlayer spacings and well‐defined nano‐capillaries. This allows electrolyte ions to diffuse into the interlayer space of rGO‐LDH. While this largely increases the number of accessible surface areas, the longer ion pathways within the interlayers naturally result in a longer diffusion time, which is reflected in the higher relaxation time constant. Such a structural difference is supported by MICP results as discussed in the previous section. Furthermore, the rGO‐LDH electrode has a real capacitance of 204 F g^−1^ at 100 mHz (Figure 4g), nearly four times higher than the rGO electrode at 59 F ^−1^g. Such high real capacitance at low frequency, typical of EDL behavior, further suggests that electrolyte ions can penetrate deeper into the electrode material.

Herein, the electrochemical measurements show that rGO‐LDH exhibits significantly enhanced capacitive performance compared to pure rGO. The intercalation of MgAl‐LDH nanoparticles as spacers in rGO largely prevents the restacking of adjacent layers. This improves both the electrolyte accessibility within the material's porous structure and the effective accessible surface area, leading to higher charge storage capacity (Figure 4h). The highly wrinkled surface, as shown in electron microscopy images, and the accessible nano‐porous structure of rGO‐LDH via MICP provide strong evidence supporting our hypothesis. These results also align with the notion that the improvements in energy storage properties of rGO‐LDH, compared with rGO, are mainly due to structural enhancements in the materials, supported by the electrochemical profiles discussed above. Furthermore, the hydrophilicity and self‐ion exchange properties of LDH nanoparticles further promote contact between the electrolyte and electrode surface.^[^ ^54^, ^55^, ^56^ ^]^ These intrinsic properties significantly enhance ion diffusion and kinetics in rGO‐LDH.

### 2.4 rGO‐LDH Supercapacitor

**Figure 5:**
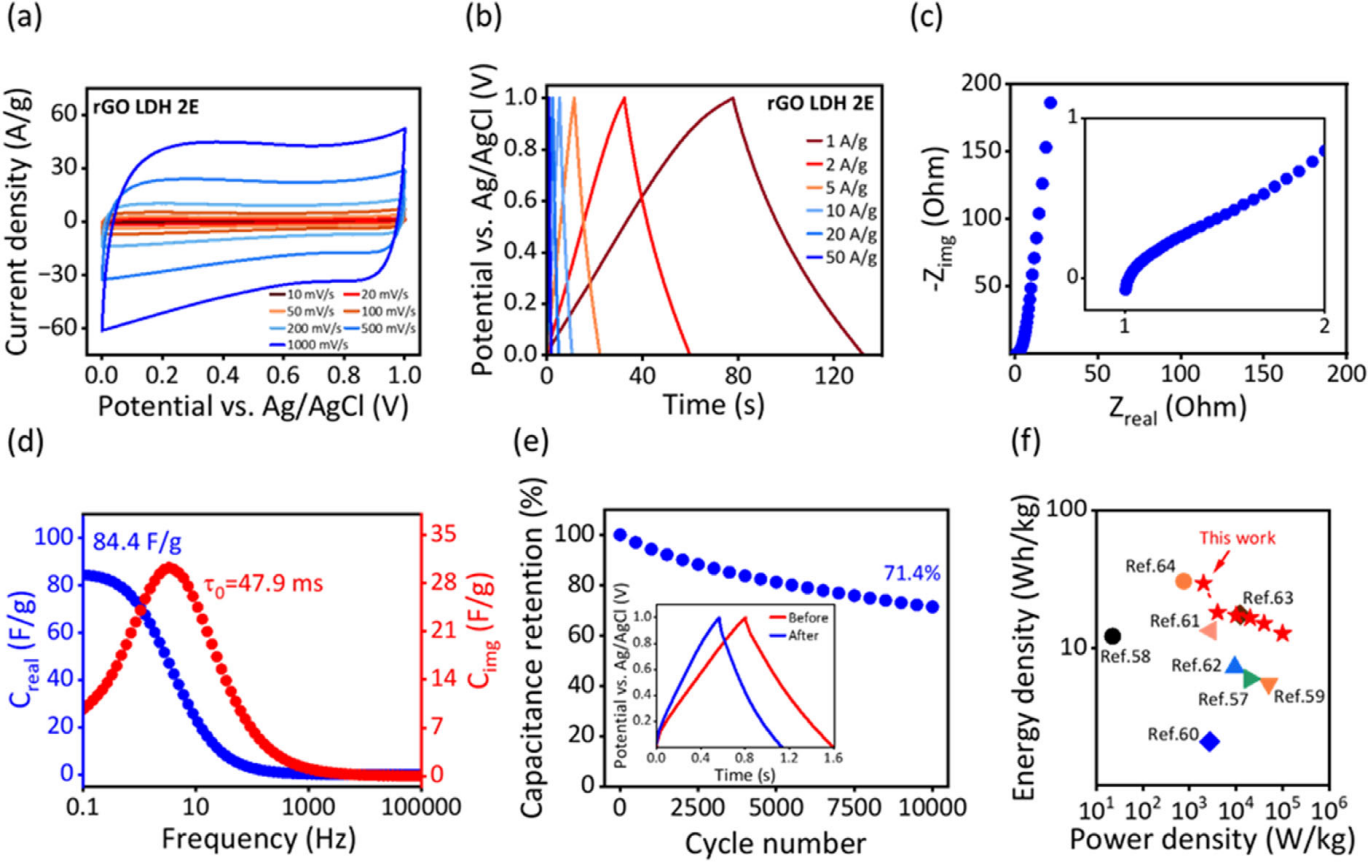
Electrochemical profiles of rGO and rGO‐LDH in two electrode system. a) Cyclic voltammetry (CV) measurements within the working potential range from 0 to 1 V with various scan rate from 10 to 1000 mV s^−1^. b) Galvanostatic charge–discharge (GCD) measurements at various current densities from 1 to 50 A g^−1^. c) Nyquist plots with an inset diagram illustrate the magnified image of Zreal from 1 to 2 Ω. d) Bode plots showing real and imaginary capacitance vs frequency. e) Cycling stability test after 10 000 cycles with a current density of 50 A g^−1^. The inset diagram shows the GCD curve of the first and the last cycle. f) Ragone plot comparing the energy and power densities of the electrodes in this work with the reported literature.^[^ ^57^, ^58^, ^59^, ^60^, ^61^, ^62^, ^63^, ^64^ ^]^ The detailed data of f) are shown in Table S1 of Note S7 in Supporting Information.

We fabricated a symmetrical two‐electrode system using rGO‐LDH to explore its potential in supercapacitor devices (**Figure** **5**). The CV curves at various scan rates (Figure 5a) display a nearly rectangular shape, confirming the ideal EDLC behavior observed previously in a three‐electrode system. In Figure 5b, the GCD profiles are almost symmetric and nearly linear, demonstrating promising charge/discharge reversibility and capacitive characteristics even at higher current densities. The rGO‐LDH electrodes achieve a specific capacitance of 212.8 F g^−1^ at a current density of 1 A g^−1^. The Nyquist plot in Figure 5c shows a near‐vertical slope in the low‐frequency region, indicating capacitive behavior. The low ESR of ≈1.2 Ω suggests minimal internal transport resistance for electrolyte ions, which is crucial for high capacitive performance. The phase angle approaching −90 ° in Figure 5d further confirms the EDL performance. The relaxation time constant of the electrodes is ≈47.9 ms, significantly lower than that of activated carbon‐based devices (≈1s).^[^ ^65^, ^66^ ^]^ Figure 5e displays the cycle stability of rGO‐LDH electrodes under a high current density of 50 A g^−1^. After 10 000 charge‐discharge cycles, the electrodes retain ≈71.4% of their initial capacitance. The relatively low cycle retention may be attributed to two main reasons. First, structural degradation and partial reconstruction of LDH nanosheets in the acidic electrolyte may induce gradual restacking of rGO laminates. Such a phenomenon could result in the decrease of effective surface area, further leading to a reduction in specific capacitance after cycling. Second, a high working current density of 50 A g^−1^ may introduce significant mechanical and structural stress to the working electrodes. This may cause partial detachment of rGO‐LDH from the current collector, thus leading to reduced performance. Nevertheless, despite these challenges, our device still maintains ≈70% of its initial capacitance after cycling, indicating considerable structural integrity. This may be due to the rGO laminates acting as a protective scaffold that confines LDH nanosheets in between and reduces their direct exposure to the acidic electrolyte, therefore slowing the degradation of LDH nanosheets. The material stability is comparable to state‐of‐the‐art studies^[^ ^57^, ^58^, ^59^, ^60^, ^61^, ^62^, ^63^, ^64^ ^]^ (Note S7, Supporting Information). Additionally, we assessed the power and energy density of our electrodes, shown in Figure 5f. The rGO‐LDH electrodes exhibit a power density of 100 kW kg^−1^ with an energy density of 12.78 Wh kg^−1^, which decreases to 2 kW kg^−1^ as the energy density increases to 29.6 Wh kg^−1^. These findings support previous results, indicating that the rGO‐LDH electrode benefits from enhanced electrolyte access, improved ion mobility, and reduced resistance, owing to the key role of LDH nanosheets as spacers. The improved laminated structure of rGO after intercalation leads to strong capacitive performance, making rGO‐LDH a promising material for high‐performance supercapacitors.

## 3 Conclusion

To summarize, we proposed a straightforward method to enhance the electrochemical energy storage capabilities of reduced graphene oxide. Based on the intercalation of magnesium‐aluminum layered double hydroxide (MgAl‐LDH) into rGO laminates via electrostatic self‐assembly, we synthesized an rGO‐LDH hybrid and investigated its electrochemical profiles and potential for supercapacitor applications. The positively charged LDH nanosheets interact strongly with the negatively charged GO surface in aqueous solution, establishing the hybrid laminated structure. Through freeze casting and thermal treatment, the hybrid GO‐LDH was reduced to rGO‐LDH, preventing self‐stacking. This results in a highly porous structure with electrolyte‐accessible nanocapillaries, which substantially increases the effective surface area and pathways for rapid ion transport.

The synthesized rGO‐LDH electrode demonstrated a superior specific capacitance of up to 410 F g^−1^, along with low internal resistance and high‐rate capability. The rGO‐LDH hybrids show an energy density of up to 30 Wh kg^−1^ and an ultrahigh power density reaching 100 kw kg^−1^ under high current density in symmetrical devices. The results indicate that intercalation of non‐redox‐active LDH nanosheets is a promising strategy for constructing the microstructure of rGO to prevent self‐stacking and further improve electrochemical energy storage performance. Our research offers a promising approach for designing next‐generation energy storage materials with enhanced efficiency and capabilities, laying the foundation for extension to other layered 2D materials for broader applications in supercapacitors, batteries, and beyond technologies.

## Conflict of Interest

The authors declare no conflict of interest.

## Supporting information



Supporting Information

## Data Availability

The data that support the findings of this study are available from the corresponding author upon reasonable request.
