# Supplementary material for: Electrostatically Induced Intercalation of Layered Double Hydroxide in Graphene Oxide for Enhanced Electrochemical Energy Storage
Source: Adv Sci (Weinh). 2025 Oct 5;12(48):e15923. doi: 10.1002/advs.202515923 (PMC12752635; doi:10.1002/advs.202515923)
Supplement: Supplementary file 1 — Supporting Information [file ADVS-12-e15923-s001.docx]

**Supporting information**

Electrostatically Induced Intercalation of Layered Double Hydroxide in Graphene Oxide for Enhanced Electrochemical Energy Storage

Xiaojun Ren^1^, Tongxi Lin^1,^ *, Bing Sun^2^, Zeno Rizqi Ramadhan^3^*,* Hang Yin^4^, Furqan Hussain^4^, Quanbin Dai^2^, Richard Tilley^3^, Liming Dai^2^, Zi Gu^2,^ *, Rakesh Joshi ^1,^ *

^1^ School of Materials Science and Engineering, University of New South Wales Sydney, NSW 2052, Australia

^2^ Australian Carbon Materials Centre (A-CMC), School of Chemical Engineering, University of New South Wales, Sydney, New South Wales, 2052 Australia

^3^Mark Wainwright Analytical Centre, University of New South Wales, Kensington, NSW, 2052, Australia

^4^ School of Minerals and Energy Resources Engineering, University of New South Wales, Sydney, NSW, 2052, Australia

**Email:** [tongxi.lin@student.unsw.edu.au](mailto:tongxi.lin@student.unsw.edu.au) ; [zi.gu1@unsw.edu.au](mailto:zi.gu1@unsw.edu.au); [r.joshi@unsw.edu.au](mailto:r.joshi@unsw.edu.au)

This file includes:

**I. Supplementary Note 1 to 9**

**II. Supplementary Figures S1 to S7**

**III. Supplementary Table S1-2**

**Supplementary Note 1. Statistical size distribution of LDH nanosheets**

We statistically analysed the distribution of thickness and the length in diameter of the synthesised LDH particles using atomic force microscopy (AFM), as exemplified in **Figure S1a** (25 data points)**.** The statistical data in total of 152 data points is shown in **Figure S1b**. The average length in diameter of LDH particles is around 38 nm, and the average thickness is around 3.3 nm. Our observed single layer of LDH particles has a thickness of around 1 nm, which agrees with its theoretical structure and observations in past studies. This indicates that our LDH particles are Tri-layers on average.


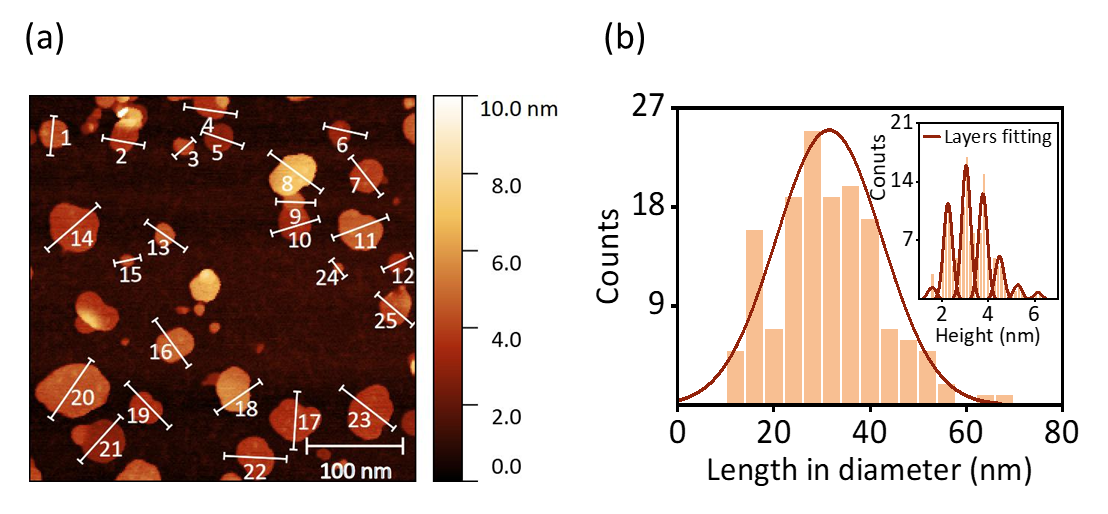


**Figure S1. (a)** Exemplary atomic force microscopy (AFM) image of LDH particles for Statistical size distribution analysis. **(b)** Statistical size distribution of length and thickness (inset diagram) for LDH particles. The brown curve in the inset diagram indicates the fitting of the layer numbers.

**Supplementary Note 2. SEM images of GO-LDH samples**

We investigated the material morphology of GO-LDH samples using scanning electron microscopy (SEM). The SEM images are shown below in **Figure S2**. We observed that both GO-LDH and rGO LDH samples have highly wrinkled surfaces; however, this is not observed on GO or rGO samples.


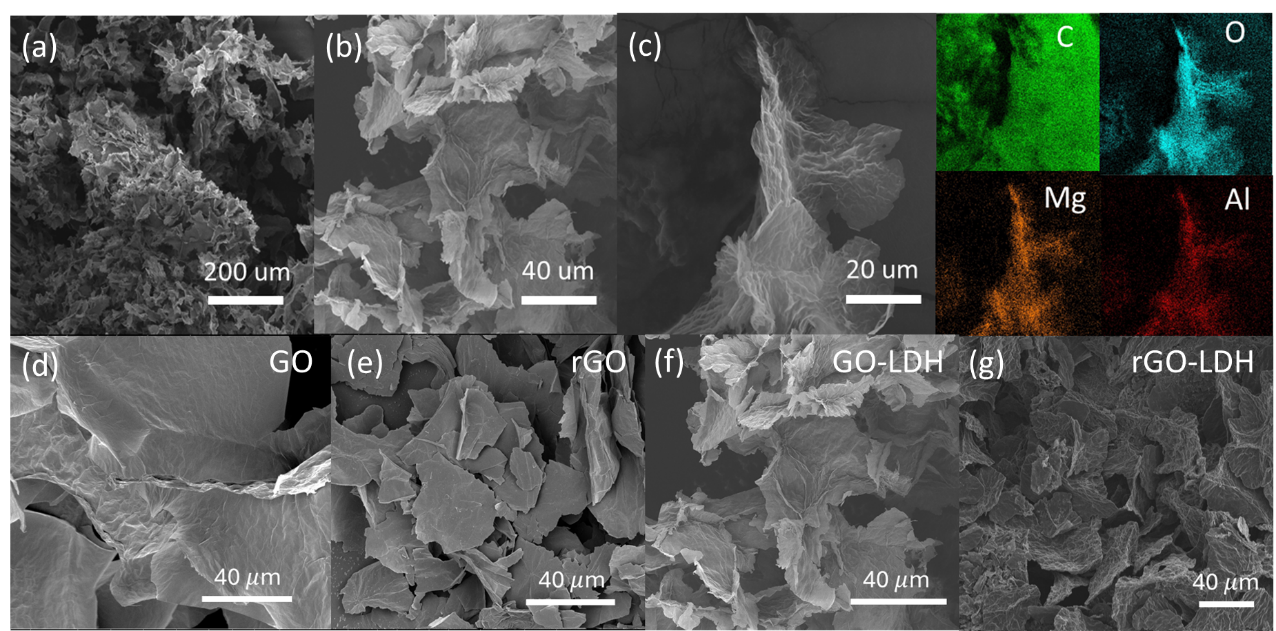


**Figure S2. (a-c)** Scanning electron microscopy (SEM) images of GO-LDH samples with energy dispersive spectroscopy (EDS) showing elemental images of carbon, oxygen, magnesium and aluminum. **(d-g)** Morphology comparison of GO (**d**), rGO (**e**)**,** GO-LDH (**f**) and rGO-LDH (**g**) within the same SEM image magnification.

**Supplementary Note 3. X-ray photoelectron spectrometry (XPS) survey scan and high-resolution spectra**

We performed the X-ray photoelectron spectrometry (XPS) survey scan for GO, rGO, LDH, GO-LDH, and rGO-LDH respectively. The XPS survey scan (**Figure S3.1 a-e**) demonstrates the elemental composition of each sample as depicted in the main text (**Figure 2a-c**).


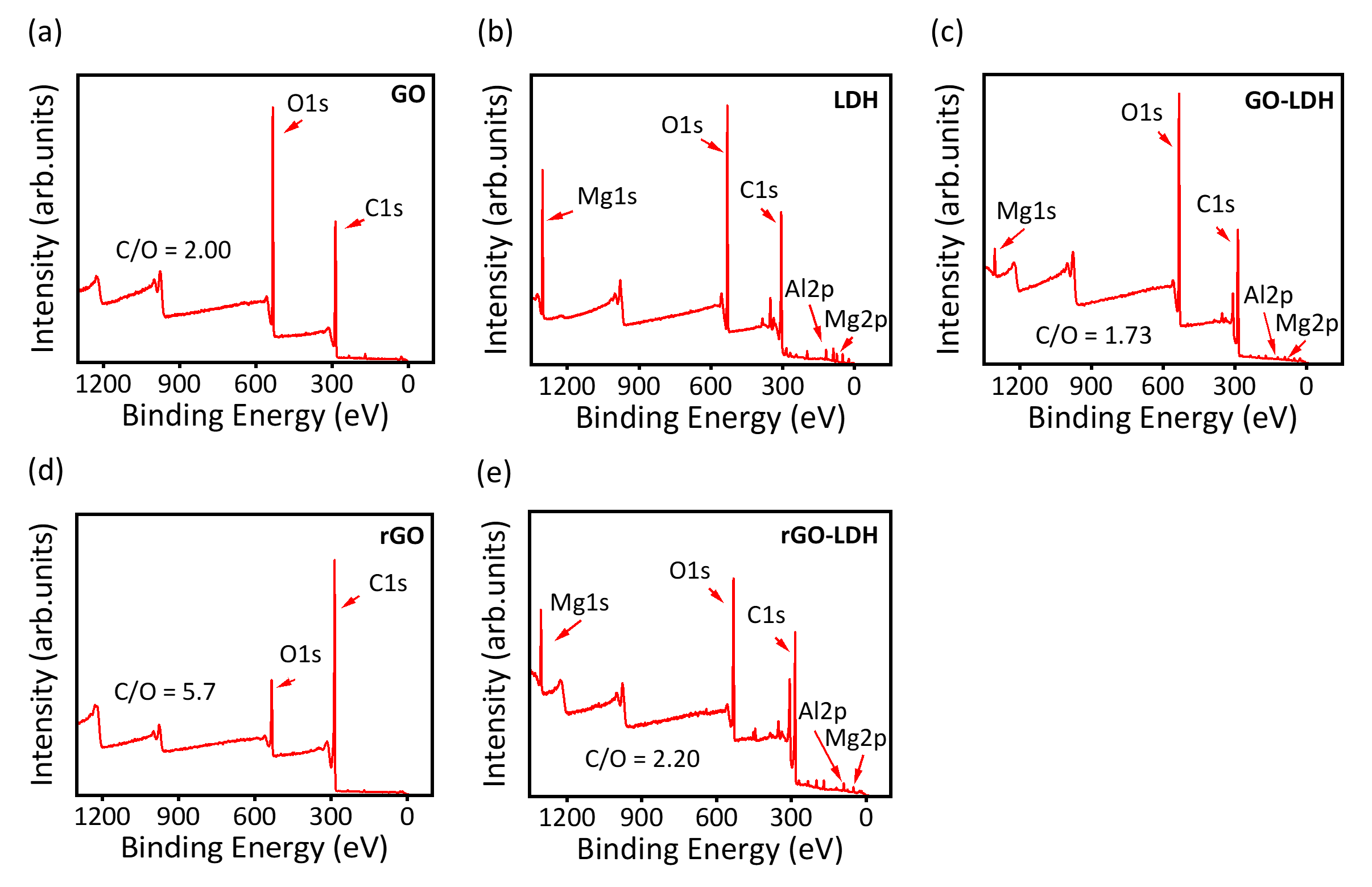


**Figure S3.1.** X-ray photoelectron spectrometry (XPS) survey of GO (**a**), LDH (**b**), GO-LDH (**c**), rGO(**d**) and rGO-LDH (**e**), showing C1s, O1s, Mg1s, Mg2p and Al2p peaks with carbon/oxygen (C/O) ratio.

We further analyzed the C1s, O1s, Mg1s and Al2p peaks with fitted curves of various bonds as shown in **Figure 3.2**.


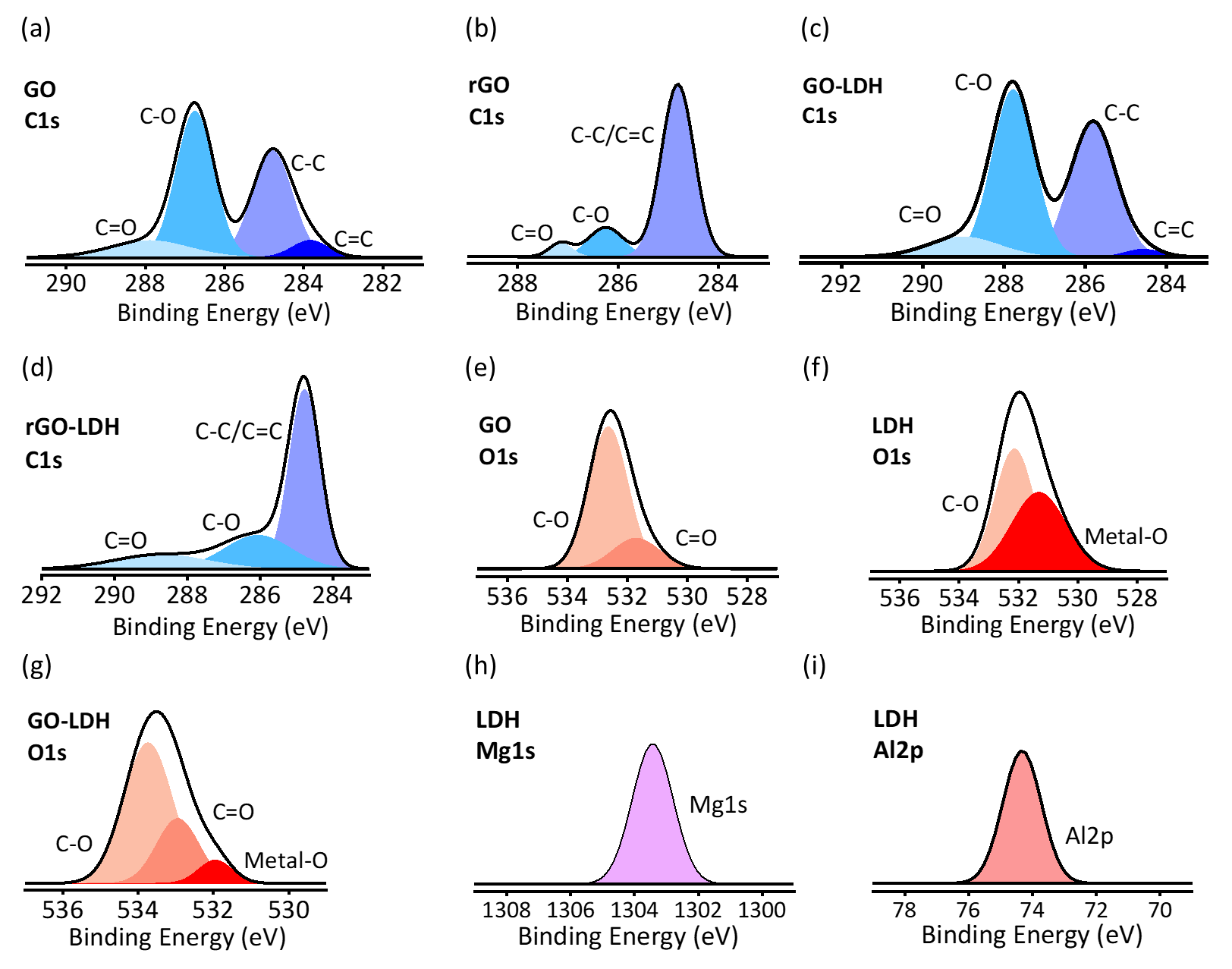


**Figure S3.2.(a-d)** C1s peak of GO, rGO, GO-LDH and rGO-LDH with peak fitting of C=C/C-C bond at ~284.5 eV, C-O bond at ~286 eV, C=O bond at ~288 eV and O-C=O at ~289 eV. **(e-g)** O1s XPS spectra of LDH, GO and GO-LDH, with peak fitting of organic C=O bond at ~531.8 eV, organic C-O bond at ~533 eV and Metal-O bond at ~530.7 eV. **(h)** Mg1s spectra of LDH with peak fitting of Mg1s at ~1303.7 e. **(i)** Al2p spectra of LDH with peak fitting of Al2p at ~1303.7 eV.

**Supplementary Note 4. Thermal stability of LDH nanosheets**

We confirmed the thermal stability of LDH particles through XRD measurements. LDH particles exhibit similar XRD patterns (**Figure S4**) before and after 10 hours of thermal treatment at 300 degrees. This suggests that no significant change in the LDH structure occurs after heating.


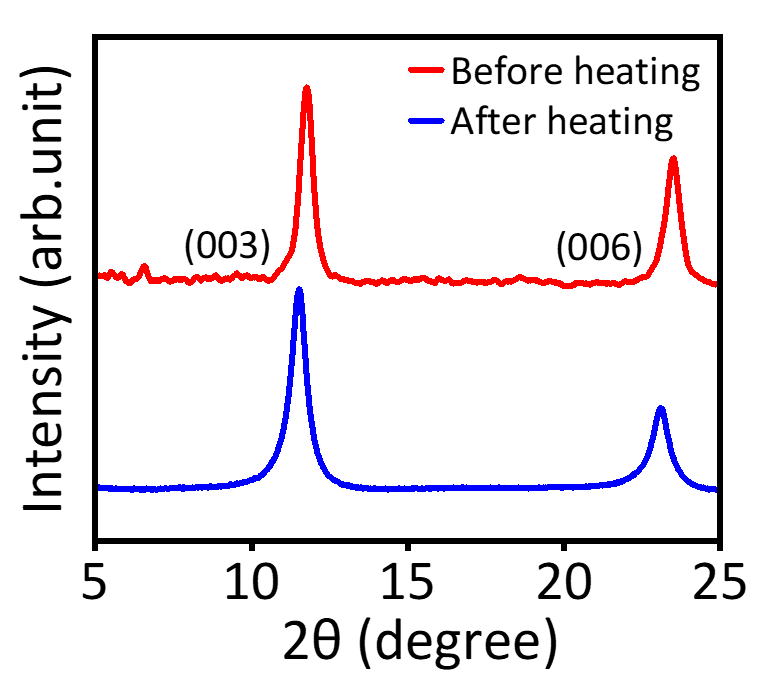


**Figure S4.** XRD patterns of LDH particles before (red curve) and after (blue curve) thermal treatment at 300 degrees.

**Supplementary Note 5. Extensive electrochemical profiles of LDH, rGO and rGO-LDH in three-electrode system**

We measured the cyclic voltammetry (CV) over a scan rate range of 10 to 1000 mV/s and conducted galvanostatic charge-discharge (GCD) tests at current densities ranging from 1 to 50 A/g for both rGO and rGO-LDH, as shown in **Figure S5**.


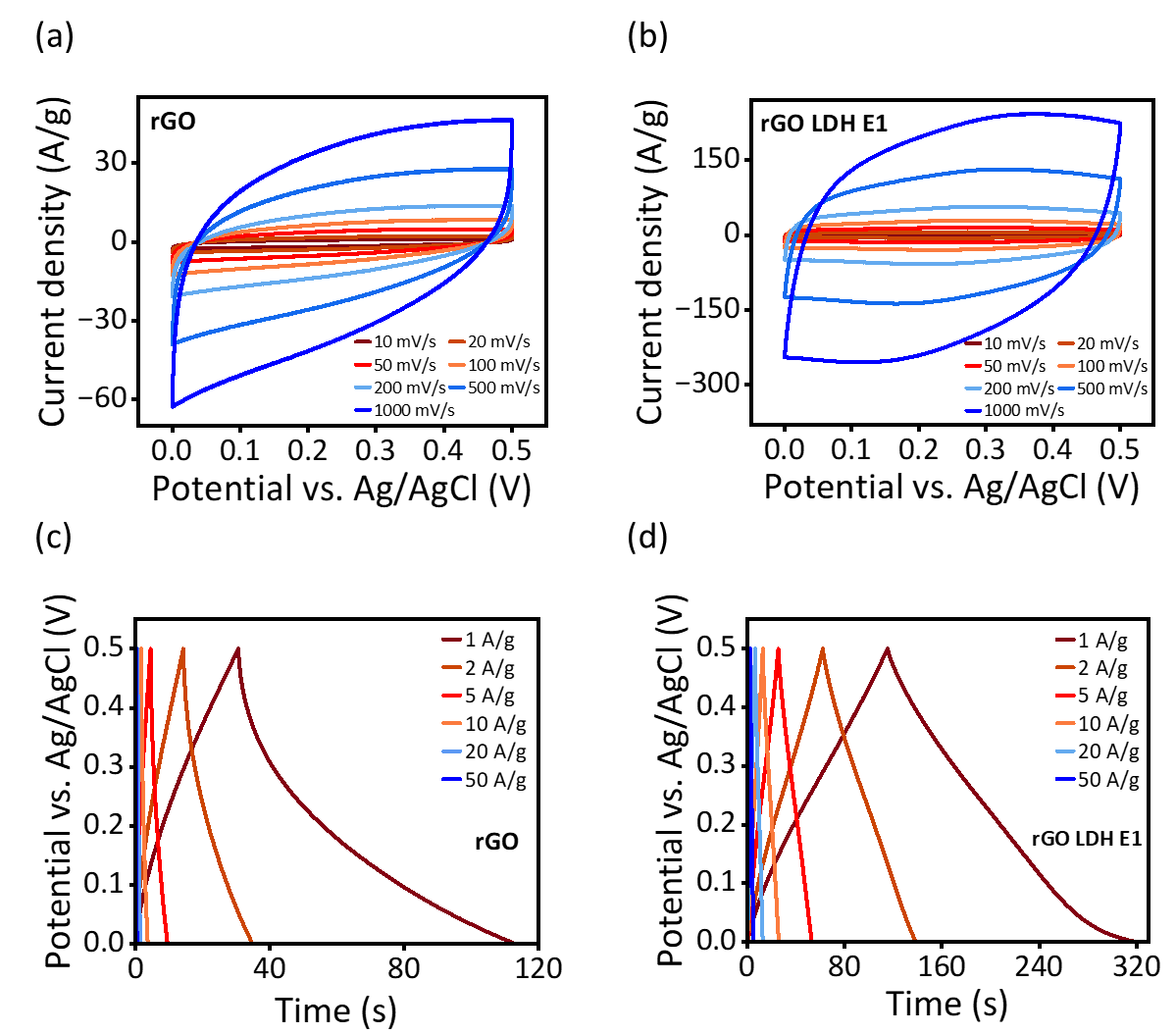


**Figure S5.1. (a-b)** Cyclic voltammetry (CV) measurements of rGO (**a**) and rGO-LDH(**b**) within the range of potential from 0 to 0.5V with various scan rates from 10 to 1000 mV/s. **(c-d)** Galvanostatic charge-discharge (GCD) tests of rGO (**c**) and rGO-LDH (**d**) with various current densities from 1 to 50 A/g.

We also measured the cyclic voltammetry (CV) for pure LDH nanoparticles at the scan rate of 200 mV/s (**Figure S5.2**). We observed that pure LDH has very limited capacitance contribution with two significant redox peaks at around 0.1 and 0.3V. This agrees with our studies that LDH particles mainly play the role of spacers rather than contributing capacitance due to their intrinsic materials composition.


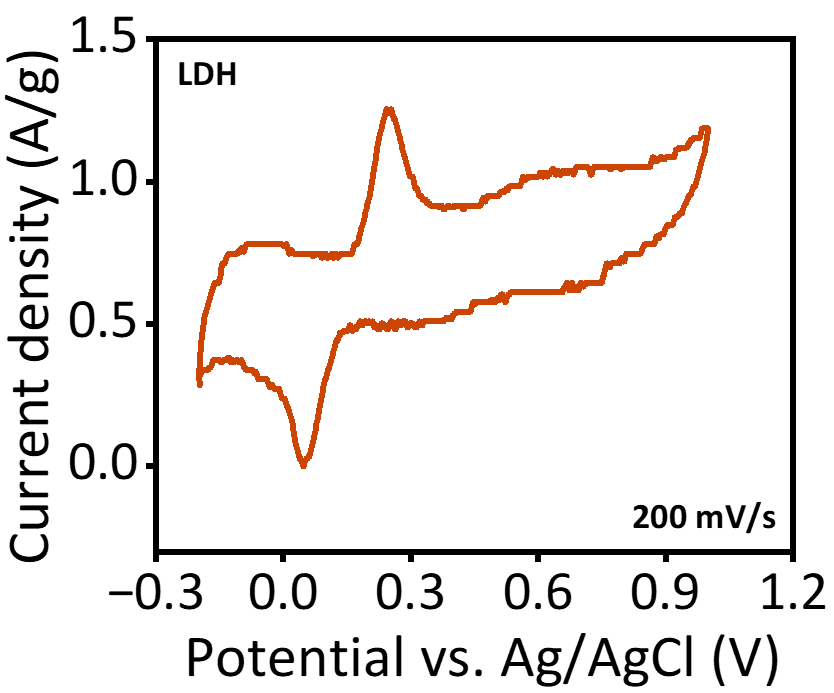


**Figure S5.2.** Cyclic voltammetry (CV) measurements of pure LDH nanoparticles within the range of potential from -0.2 to 1 V at the scan rate of 200 mV/s.

**Supplementary Note 6. Capacitance contribution of rGO-LDH and rGO electrodes from the CV curves**

We investigated the capacitance contribution of the rGO-LDH and rGO electrodes using CV curves based on Dunn’s method^[1,2]^. This approach helps us understand the capacitive and diffusion-controlled processes respectively that occur on the electrodes. This method can be expressed with the equation,

$$\begin{aligned} i\left( V \right)=k_{1}v+k_{2}v^{\frac{1}{2}}\#\left( S6.1 \right) \end{aligned}$$

Where $i\left( V \right)$ represents the current density (A/g), $k_{1}$ and $k_{2}$ represents the capacitive and diffusion-controlled factor respectively, and $v$ represents the scan rate (mV/s) of the CV measurements.

This formula can be further derived to,

$$\begin{aligned} \frac{i\left( V \right)}{v^{\frac{1}{2}}}=k_{1}v^{\frac{1}{2}}+k_{2}\#\left( S6.2 \right) \end{aligned}$$

From equation (S6.2), a plot of $\frac{i\left( V \right)}{v^{\frac{1}{2}}}$ ***vs.*** $v^{\frac{1}{2}}$ can be drawn via linear fitting. Herein, $k_{1}$is the slope and $k_{2}$ is the interception of this curve, respectively. With this, the capacitive contribution $k_{1}v$ can be calculated at various voltages (V).

In this analysis, 1000 data points of current density are calculated within the potential window of 0 to 0.5V, with a homogeneous distribution of voltage. The enclosed area represents the capacitive contribution, and the remaining area from the CV curve indicates the diffusion-controlled contribution. The comparison of the contributions of rGO-LDH and rGO electrodes at various scan rates (10, 20, 50 and 100 mV/s) is shown in **Figure S6.1a-b**.


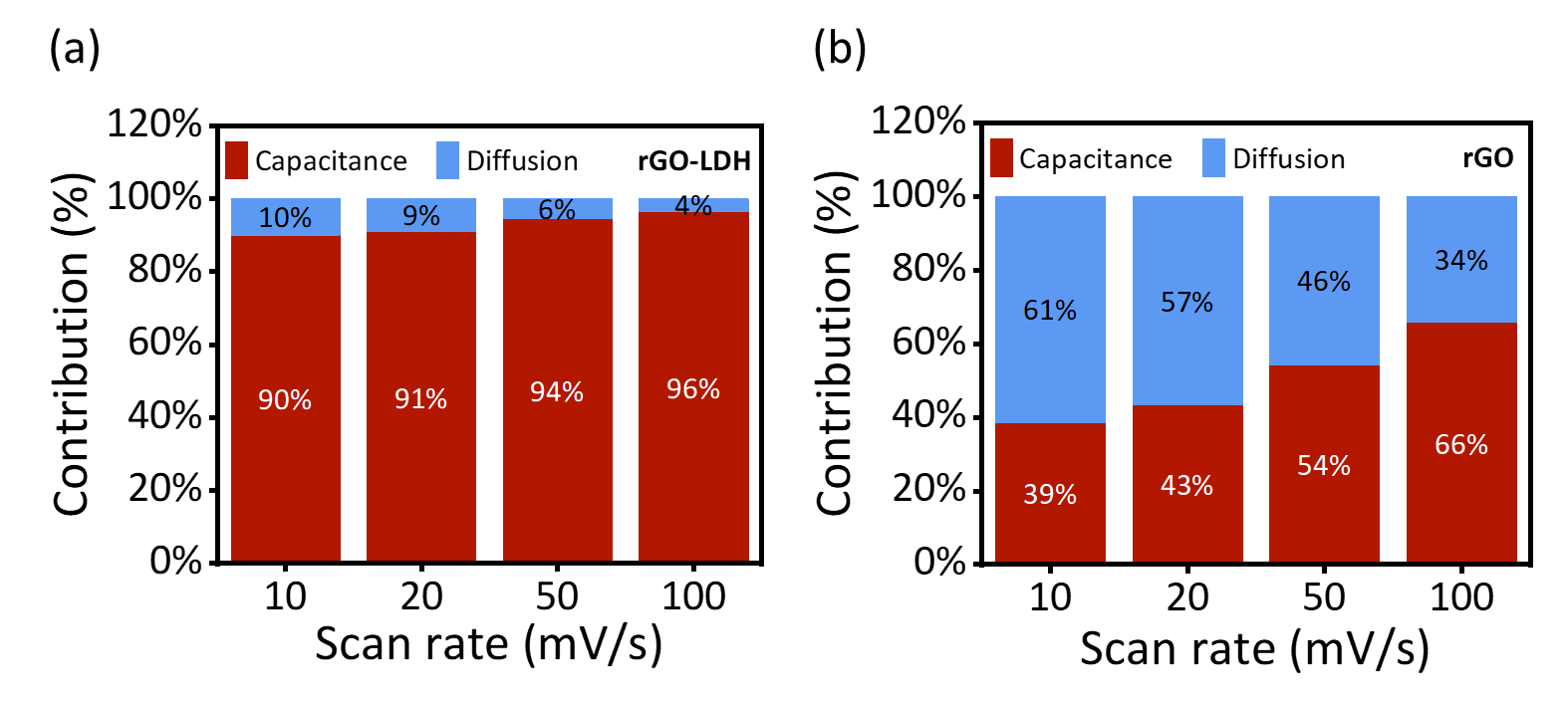


**Figure S6.1. (a-b)** Comparison of the capacitance and diffusion contribution with increasing scan rate from 10 to 100 mV/s for rGO-LDH (**a**) and rGO(**b**).

Each individual CV curve with calculated contribution area is shown in **Figure S6.2 a-d** for rGO-LDH and **Figure S6.2 e-h** for rGO respectively.


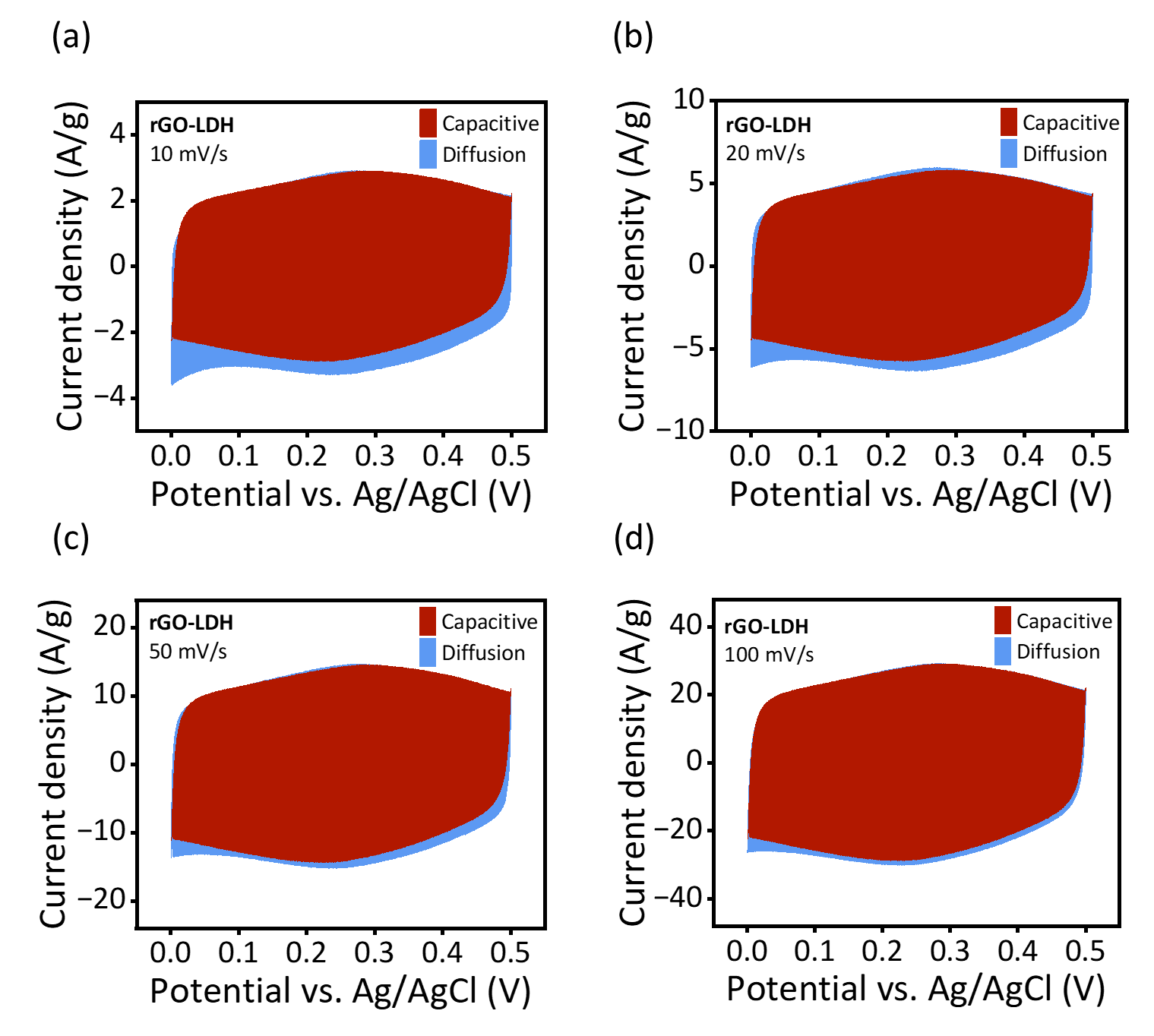


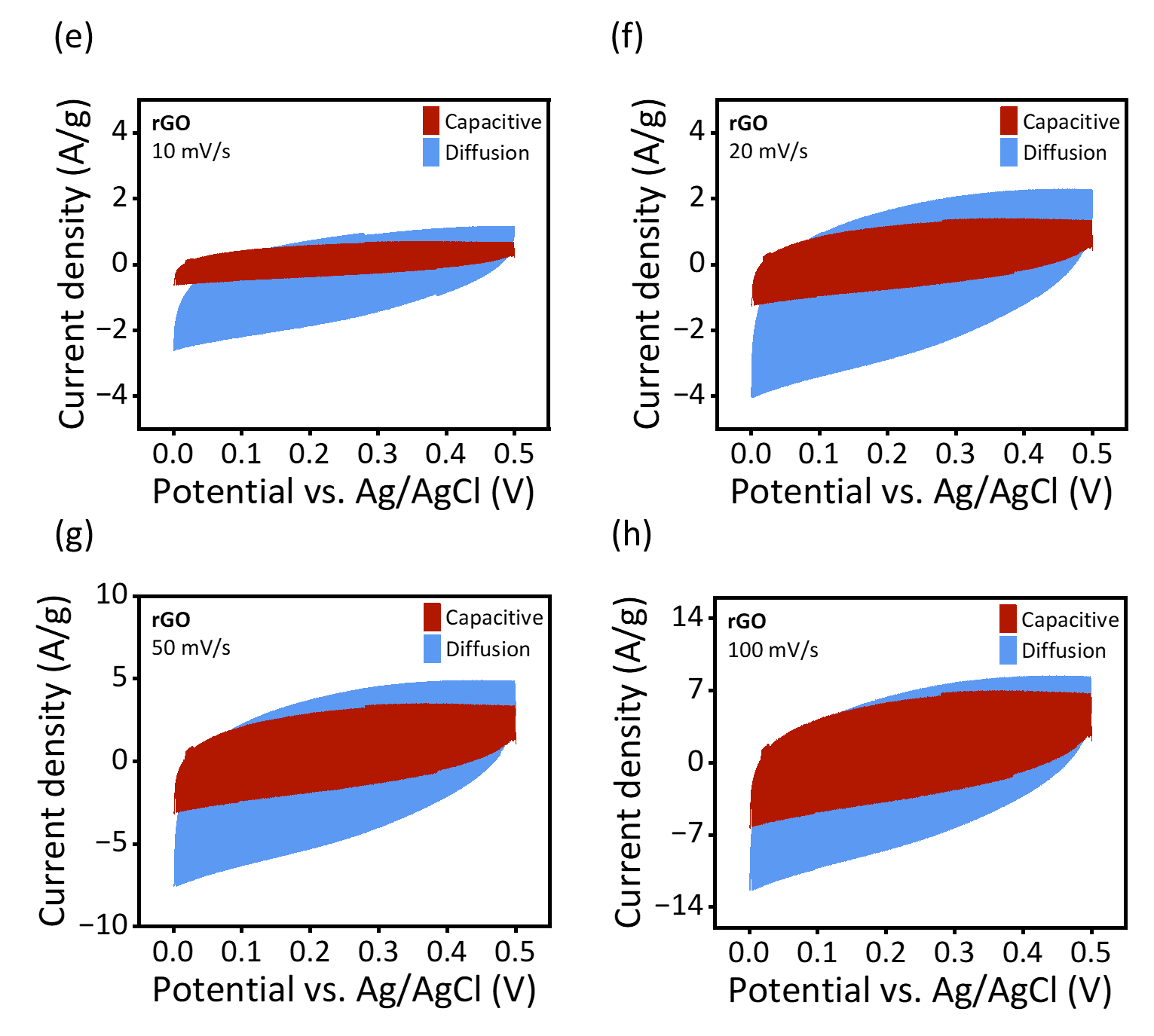


**Figure S6.2. (a-h)** Capacitance and diffusion contribution from CV curves with various scan rates (10, 20, 50 and 100 mV/s) for rGO-LDH (**a-d**) and rGO (**e-h**).

**Supplementary Note 7. Summary of** **power and energy density performance of various state-of-art reported carbon-based supercapacitors**

**Table S1.** Power and energy density performance of various state-of-art reported carbon-based supercapacitors versus this work.

| **Sample** | **Energy density (Wh/kg)** | **Power density (W/kg)** | **Electrolyte** | **Ref.** | |
| --- | --- | --- | --- | --- | --- |
| WC@MnO2 | 12.2 | 22.3 | 6M KOH | | ^[1]^ |
| Doped carbon nanoflakes | 7.3 | 9360 | 2M KOH | | ^[2]^ |
| Defect rich porous carbon | 5.54 | 50000 | 6M KOH | | ^[3]^ |
| B/N co-doped carbon | 2.1 | 2700 | 1M H_2_SO_4_ | | ^[4]^ |
| NiS_2_ hybrid carbon | 13.4 | 2804 | PVA/KOH | | ^[5]^ |
| Heteroatom-doped porous carbon | 6 | 19600 | 6M KOH | | ^[6]^ |
| Nitrogen-doped carbon | 17.5 | 12300 | 6M KOH | | ^[7]^ |
| NCM-NS-CNT30 | 30.6 | 750 | 6M KOH | | ^[8]^ |
| rGO-LDH | 29.5 | 2000 | 1M H_2_SO_4_ | | This work |
|  | 18.2 | 4000 |  |  |  |
|  | 17.4 | 10000 |  |  |  |
|  | 16.6 | 20000 |  |  |  |
|  | 15.1 | 40000 |  |  |  |
|  | 12.8 | 100000 |  |  |  |

**Table S2.** Cycle performance of various state-of-art reported carbon-based supercapacitors versus this work.

| **Sample** | **Cycle numbers** | **Cycle stability** | **Current density (A/g)** | **Ref.** | |
| --- | --- | --- | --- | --- | --- |
| WC@MnO2 | 10000 | 75.2% | 2.3 | | ^[1]^ |
| Doped carbon nanoflakes | 10000 | 93% | 10 | | ^[2]^ |
| Defect rich porous carbon | 20000 | 98% | - | | ^[3]^ |
| B/N co-doped carbon | 30000 | 99% | 10 | | ^[4]^ |
| NiS_2_ hybrid carbon | 8000 | 91% | 3 | | ^[5]^ |
| Heteroatom-doped porous carbon | 5000 | 80% | - | | ^[6]^ |
| Nitrogen-doped carbon | 20000 | 87.4% | 50 | | ^[7]^ |
| NCM-NS-CNT30 | 10000 | 82.3% | 1 | | ^[8]^ |
| rGO- LDH | 10000 | 71.4% | 50 | | This work |

**Supplementary Note 8 Experimental set-up of electrochemical measurements**


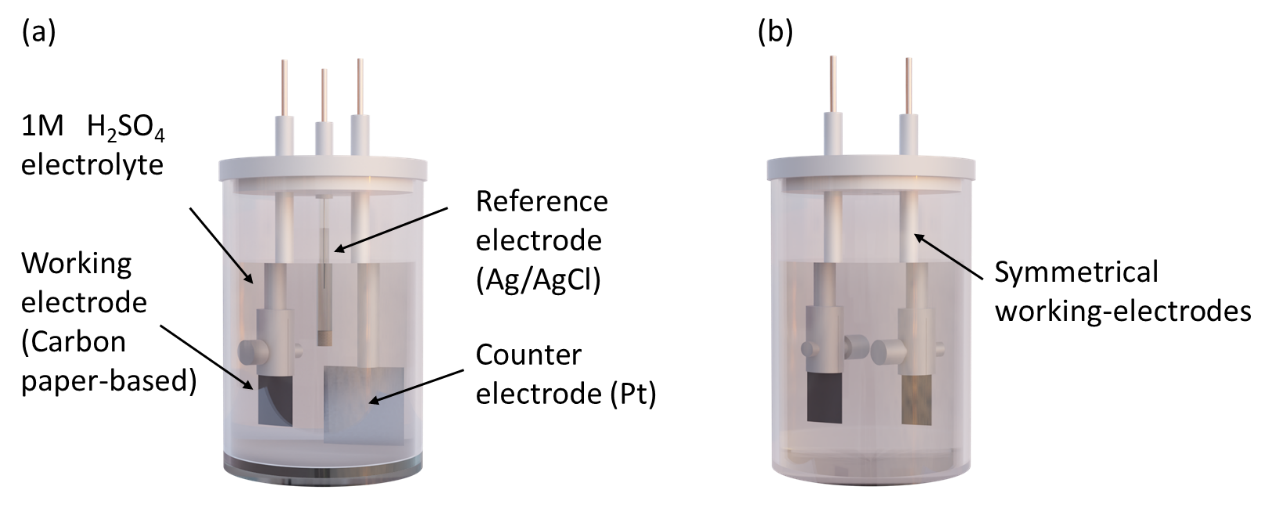


**Figure S7 (a-b)** Electrochemical working cell of three-electrode system (**a**) and two-electrode system (**b**).

**Supplementary Note 9 Experimental methods and electrochemical calculations**

**Materials**

Graphene oxide solution (1.5 wt%) is supplied by NiSiNa Materials Japan. The obtained GO solution was then prepared in de-ionised (DI) water at a concentration of 10 mg/mL, followed by 30 minutes of sonication. MgCl_2_·6H_2_O (99%), AlCl_3_·6H_2_O (99%), and NaOH (98%) were purchased from Sigma-Aldrich. Anhydrous ethanol (100%) was obtained from Chem-Supply AU. Silicon wafers were purchased from Graphene Supermarket Ptd. KOH (98%) powder was purchased from Chem-Supply AU and prepared as a 1 M solution without further treatment.

**Synthesis of rGO-LDH hybrid materials**

LDH nanoparticles were prepared using the traditional co-precipitation method. A mixture of 10 ml MgCl_2_·6H_2_O (3 mmol) and AlCl_3_·6H_2_O (1 mmol) was added to 40 ml of NaOH solution (6 mmol) with continuous stirring for 10 minutes. The resulting solution was centrifuged three times and re-dispersed in water. LDH particles were then combined with GO stock solution at the desired ratio, followed by 15 minutes of stirring and one hour of sonication. The GO-LDH mixture was rinsed with water and freeze-dried at -60 °C to obtain sponge-like powders, which were subsequently heated at 300 °C for 10 hours in a muffle furnace to induce reduction. The resulting rGO-LDH powder was dispersed in anhydrous ethanol at a concentration of 5 mg/ml to prepare a stock solution.

**Material characterizations**

The physicochemical characterisation of the synthesised samples included various analyses. The structure, including the thickness and size of the LDH samples, was investigated by drop casting the sample solution onto a silicon wafer and analysed using an atomic force microscope (Bruker Dimension ICON SPM). The chemical composition of the synthesised samples was confirmed with an X-ray photoelectron spectrometer (Thermo ESCALAB250i XPS). The interlayer spacing of the synthesised materials was analysed through X-ray diffraction (XRD, PANalytical Empyrean IV) at 45 kV 40 mA with Cu Kα radiation (λ = 0.154 nm). The zeta potential and particle size of the aggregated solution samples were investigated using a dynamic light scattering system (Zetasizer Ultra ZS) under mass ratio gradient. The porosity and surface area of the samples were measured with a Mercury injection capillary pressure (MICP) analyser (PM 33-GT-12, POREMASTER GT). The surface morphology and microstructure of the synthesised samples were examined with a field-emission scanning electron microscope (FEI Nova NanoSEM 230 FE-SEM) equipped with an EDS detector (Bruker SDD-EDS) to confirm the elemental composition. The nanostructure was studied using transmission electron microscopy (TEM, JEOL JEM-F200 Multi-Purpose FEG-S/TEM), combined with an EDS detector.

**Fabrication of the working electrode**

The working electrode for supercapacitance studies was prepared using Carbon Fibre Paper (CFP). Super P Carbon Black (5 mg/ml in ethanol) was added to the rGO-LDH stock solution as a conductor, following a 1:10 ratio to obtain an ink-like slurry. The slurry was sonicated for 1 hour to ensure thorough dispersion. Approximately 0.1 mg of the slurry was homogenously drop-cast onto the hydrophilic CFP with a surface area of about 1 cm^2^. The mass loading was verified by weighing the change in mass of the CFP using an analytical balance with a detection limit of 0.01 mg.

**Electrochemical characterizations**

Electrochemical performances were conducted using the Bio-Logic VSP-300 Multichannel Potentiostat (Pennsylvania, USA), employing both two-electrode and three-electrode systems in a 1 M electrolyte solution. The experimental setup is detailed in **Supplementary Note 8**. An Ag/AgCl electrode served as the reference electrode, while a 6.25 cm² platinum (Pt) plate was used as the counter electrode. In the two-electrode configuration, cyclic voltammetry (CV) curves were recorded at scan rates ranging from 10 mV/s to 1000 mV/s within a potential window of 0 V to 1 V. Galvanostatic charge-discharge (GCD) measurements were performed at current densities from 1 A/g to 50 A/g over the same potential range. Electrochemical impedance spectroscopy (EIS) measurements were carried out at open circuit potential using a sinusoidal signal with a 5 mV amplitude, spanning frequencies from 100 kHz to 100 mHz. Cycling stability tests involved continuous long-term GCD experiments over 10000 cycles.

The electrochemical calculations are shown below, based on the reported literature ^[9–11]^.

The capacitance of the electrodes in a three-electrode system was calculated based on the galvanostatic charge-discharge curve, using the formula,

$$C_{single}=\frac{i\Delta t}{\Delta V}$$

where $C_{single}$ represents the capacitance (F) of the working electrode, $i$ represents the current (A), $\Delta t$ indicates the consumed time for one complete charge-discharge cycle of the working electrode, and $\Delta V$ describes the working voltage range (V) of the working electrode.

The specific capacitance (F/g) of the supercapacitance ($C_{sc}$) can be calculated as,

$$C_{sc}=\frac{C_{single}}{m_{single}}$$

where $m_{single}$represents the mass loading of the working electrode.

The rate capability (R) of the electrodes in a three-electrode system was calculated based on the specific capacitance at different current densities using the formula.

$$R=\frac{{C'}_{sc}}{C_{sc}}\times100\%$$

where $C_{sc}$ and ${C'}_{sc}$ represents the specific capacitance (F/g) of the working electrode under two different current density, which ${C'}_{sc}$ basically is the specific capacitance under high current density and $C_{sc}$ is which under low current density.

The capacitance (F) of the supercapacitor ($C_{two}$) in a two-electrode system was also calculated based on the galvanostatic charge-discharge curve as mentioned. Here, the supercapacitor consists of two electrodes, a positive and a negative one. Assuming they are equal within the system and connected in series in a symmetrical supercapacitor, the capacitance of the supercapacitor,

$$\frac{1}{C_{two}}=\frac{1}{C_{positive}}+\frac{1}{C_{negative}}$$

also, the mass loading of one electrode in the supercapacitor could be assumed as half of the whole system,

$$m_{two}=2 m_{single}$$

Where $m_{two}$ represents the mass loading of the supercapacitor and $m_{single}$represents the mass loading of one single electrode of the supercapacitor as ab0ve.

Therefore, based on these two equations, the specific capacitance (F/g) of the supercapacitance (${C^{*}}_{sc}$) could be derived as,

$${C^{*}}_{sc}= \frac{C_{two}}{m_{two}}=\frac{C_{single}}{m_{single}}=\frac{{4C}_{two}}{m_{single}}$$

It is worth to be mentioned here that in theoretical conditions, the specific capacitance (F/g) of the supercapacitance $C_{sc}$ and ${C^{*}}_{sc}$ would be the same in both two-electrode and three-electrode system.

Within a two-electrode system, the whole system could be considered as a device. Thus, the specific energy density ($E_{sc}$) of the device can be calculated as follow with unit of Wh/kg,

$$E_{sc}=\frac{1}{2} {C^{*}}_{sc} {(\Delta V-V_{IR})}^{2} \times\frac{1}{3600}$$

where $\Delta V$ represents the working potential range (V), $V_{IR}$ represents the IR drop during the beginning of the discharge procedure in the galvanostatic charge-discharge cycles.

With the energy density, the power density ($P_{sc}$) of the device could be further calculated with unit of W/kg,

$$P_{sc}=\frac{E_{sc}\times3600}{t_{discharge}}$$

Where $t_{discharge}$ (s) represents the discharge time during the galvanostatic charge-discharge cycles.

In electrochemical impedance spectroscopy (EIS) curves, the measured Bode plots indicates the time constant ($\tau_{0}$), using following formula to calculate,

$$\tau_{0}=\frac{1}{2\pi f_{0}}$$

where $f_{0}$ represents the frequency when phase angle equals to $-45^{\circ}$ .

To calculate the real capacitance (*C_real_*) (F/g) and the imaginary capacitance (*C_img_*) (F/g), the following formulas are used,

$$C_{real}= \frac{-Z_{img}}{2\pi f\left| Z \right|^{2}m}$$

$$C_{img}= \frac{Z_{real}}{2\pi f\left| Z \right|^{2}m}$$

Where *Z_real_* is the real part of impedance (Ω), *Z_img_* is the imaginary part of impedance (Ω), *f* is the frequency (Hz) and *m* is the loading mass of the active materials (g).

**References**

[1] L. Chen, F. Wang, Z. Tian, H. Guo, C. Cai, Q. Wu, H. Du, K. Liu, Z. Hao, S. He, G. Duan, S. Jiang, Wood‐Derived High‐Mass‐Loading MnO _2_ Composite Carbon Electrode Enabling High Energy Density and High‐Rate Supercapacitor. *Small* **2022**, *18*.

[2] N. Mao, H. Wang, Y. Sui, Y. Cui, J. Pokrzywinski, J. Shi, W. Liu, S. Chen, X. Wang, D. Mitlin, Extremely high-rate aqueous supercapacitor fabricated using doped carbon nanoflakes with large surface area and mesopores at near-commercial mass loading. *Nano Res* **2017**, *10*, 1767.

[3] L. Hou, W. Yang, B. Jiang, P. Wang, L. Yan, C. Zhang, G. Huang, F. Yang, Y. Li, Intrinsic defect-rich porous carbon nanosheets synthesized from potassium citrate toward advanced supercapacitors and microwave absorption. *Carbon N Y* **2021**, *183*, 176.

[4] J. Hao, J. Wang, S. Qin, D. Liu, Y. Li, W. Lei, B/N co-doped carbon nanosphere frameworks as high-performance electrodes for supercapacitors. *J Mater Chem A Mater* **2018**, *6*, 8053.

[5] M. Fu, Z. Zhu, W. Chen, H. Yu, R. Lv, Carbon cloth supported flower-like porous nickel-based electrodes boosting ion/charge transfer characteristics of flexible supercapacitors. *Carbon N Y* **2022**, *199*, 520.

[6] H. Wang, Y. Yuan, F. Xiong, B. Ma, J. Yang, Y. Qing, F. Chu, Y. Wu, Bidirectional pore-creating strategy towards lignin-based heteroatom-doped porous carbon for supercapacitors. *Chemical Engineering Journal* **2023**, *476*, 146640.

[7] B. Yan, L. Feng, J. Zheng, Q. Zhang, Y. Dong, Y. Ding, W. Yang, J. Han, S. Jiang, S. He, Nitrogen-doped carbon layer on cellulose derived free-standing carbon paper for high-rate supercapacitors. *Appl Surf Sci* **2023**, *608*, 155144.

[8] Y. Hao, H. Guo, L. Peng, J. Xu, J. Tian, H. Ren, W. Yang, Cobalt carbonate hydroxide-embedded in multi-layered holey nanoflower cross-linked with carbon nanotube for highly efficient supercapacitors with ultrahigh rate performance. *Electrochim Acta* **2024**, *473*, 143513.

[9] N. B. Mohamed, M. F. El‐Kady, R. B. Kaner, Macroporous Graphene Frameworks for Sensing and Supercapacitor Applications. *Adv Funct Mater* **2022**, *32*.

[10] A. Noori, M. F. El-Kady, M. S. Rahmanifar, R. B. Kaner, M. F. Mousavi, Towards establishing standard performance metrics for batteries, supercapacitors and beyond. *Chem Soc Rev* **2019**, *48*, 1272.

[11] Y. Shao, M. F. El‐Kady, C. Lin, G. Zhu, K. L. Marsh, J. Y. Hwang, Q. Zhang, Y. Li, H. Wang, R. B. Kaner, 3D Freeze‐Casting of Cellular Graphene Films for Ultrahigh‐Power‐Density Supercapacitors. *Advanced Materials* **2016**, *28*, 6719.
